# Screening and Identification of Hub Genes in the Development of Early Diabetic Kidney Disease Based on Weighted Gene Co-Expression Network Analysis

**DOI:** 10.3389/fendo.2022.883658

**Published:** 2022-06-03

**Authors:** Ran Wei, Jingtao Qiao, Di Cui, Qi Pan, Lixin Guo

**Affiliations:** ^1^ Department of Endocrinology, Peking University Fifth School of Clinical Medicine, Beijing, China; ^2^ Department of Endocrinology, Beijing Hospital, National Center of Gerontology, Institute of Geriatric Medicine, Chinese Academy of Medical Sciences, Beijing, China; ^3^ Department of Pathology, Beijing Hospital, National Center of Gerontology, Institute of Geriatric Medicine, Chinese Academy of Medical Sciences, Beijing, China

**Keywords:** bioinformatics, WGCNA, GSEA analysis, early diabetic kidney disease, hub gene

## Abstract

**Objective:**

The study aimed to screen key genes in early diabetic kidney disease (DKD) and predict their biological functions and signaling pathways using bioinformatics analysis of gene chips interrelated to early DKD in the Gene Expression Omnibus database.

**Methods:**

Gene chip data for early DKD was obtained from the Gene Expression Omnibus expression profile database. We analyzed differentially expressed genes (DEGs) between patients with early DKD and healthy controls using the R language. For the screened DEGs, we predicted the biological functions and relevant signaling pathways by enrichment analysis of Gene Ontology (GO) biological functions and Kyoto Encyclopedia of Genes and Genomes (KEGG) signaling pathways. Using the STRING database and Cytoscape software, we constructed a protein interaction network to screen hub pathogenic genes. Finally, we performed immunohistochemistry on kidney specimens from the Beijing Hospital to verify the above findings.

**Results:**

A total of 267 differential genes were obtained using GSE142025, namely, 176 upregulated and 91 downregulated genes. GO functional annotation enrichment analysis indicated that the DEGs were mainly involved in immune inflammatory response and cytokine effects. KEGG pathway analysis indicated that C-C receptor interactions and the IL-17 signaling pathway are essential for early DKD. We identified FOS, EGR1, ATF3, and JUN as hub sites of protein interactions using a protein–protein interaction network and module analysis. We performed immunohistochemistry (IHC) on five samples of early DKD and three normal samples from the Beijing Hospital to label the proteins. This demonstrated that FOS, EGR1, ATF3, and JUN in the early DKD group were significantly downregulated.

**Conclusion:**

The four hub genes FOS, EGR1, ATF3, and JUN were strongly associated with the infiltration of monocytes, M2 macrophages, and T regulatory cells in early DKD samples. We revealed that the expression of immune response or inflammatory genes was suppressed in early DKD. Meanwhile, the FOS group of low-expression genes showed that the activated biological functions included mRNA methylation, insulin receptor binding, and protein kinase A binding. These genes and pathways may serve as potential targets for treating early DKD.

## Introduction

Diabetic kidney disease (DKD) is a formidable health challenge that we are faced with. It occurs in up to 50% of patients with diabetes and is the dominant cause of end-stage renal disease (1, 2). However, microalbuminuria is the most common early clinical symptom of DKD and is usually undetected and easily ignored by patients. Following the onset of early DKD, chronic renal failure eventually develops into uremia as the disease progresses. As a result, when patients exhibit obvious symptoms, such as massive proteinuria and renal failure, most patients have progressed to advanced DKD (3–5). Screening key genes in early DKD and clarifying their biological functions is expected to predict the development of DKD as early as possible.

Glomerular endothelial dysfunction plays a crucial role in the pathogenesis of early DKD (6). Hyperglycemia induces oxidative stress, endoplasmic reticulum stress, and apoptosis in the early stages of DKD (7, 8). Inflammation and immune regulation are the fundamental mechanisms underlying the development and progression of DKD. Epigenetic contributions to inflammation and fibrogenesis occur at different regulatory levels, namely, DNA methylation and non-coding RNA modulation (9). The cytokine–cytokine (C–C) receptor interaction pathway and activated biological functions are also essential parts of the above network.

In recent years, thanks to the rapid development of bioinformatics and gene chip technology, the establishment and improvement of many disease databases have provided the theoretical basis for revealing pathogenesis and new therapeutic targets. Differentially expressed genes (DEGs) and hub genes could help us to better understand the molecular mechanisms underlying DKD progression and provide candidate targets for the diagnosis and treatment of DKD (10–14). In this study, we aimed to screen DEGs in early DKD patients compared with those in normal kidney tissue and to explore the biological functions and possible mechanisms of the DEG signaling pathway. This finding may provide a promising direction for clarifying the diagnosis and pathogenesis of early DKD.

## Materials and Methods

### Data Collection

The National Center for Biotechnology Information (NCBI) Gene Expression Omnibus (GEO) database (https://www.ncbi.nlm.nih.gov/geo/) was used to obtain early DKD relevant gene expression profile data. We obtained the original data from human kidney tissue gene chip GSE142025, which contained the data of nine normal kidney tissues and six early DKD samples, using the keyword “early diabetic nephropathy” or “early diabetic kidney disease” in the GEO database.

### Data Processing and Differential Expression Analysis

We ran the R language script to read the dataset downloaded from the GEO database and normalize the data. Our research analyzed the standardization of chip expression spectrum differences *via* R language functions and limma packages, tested correction through the Bayes method multiple, and filtered DEGs with a standard of | log2FC | >2 and *P <*0.05. We performed cluster analysis of DEGs and created a heatmap using gplots in the R language.

### GO Enrichment Analysis of Differential Genes and KEGG Pathway Analysis

We analyzed the DEGs of selected data using the DAVID database (https://david.ncifcrf.gov/) (15), according to the GO analysis of gene function annotation enrichment. Molecular functions, cellular components, and biological processes were part of the data that was analyzed (16). KEGG was used to annotate DEGs, which mainly covered gene function, biological pathways, cell localization, and signaling pathways. The KEGG signaling pathway was analyzed using the KOBAS database (17), with a screening condition of *P <*0.05.

### Differential Gene Protein Interaction Network Analysis

Protein–protein interaction (PPI) analysis and modular analysis of differential genes were performed using the STRING database (http://string-db.org/) (18) and Cytoscape software (19). We imported DEGs into the STRING database and analyzed the PPI of the differential genes. Then, we used the degree plug-in of Cytoscape software to analyze the module of results and mined the most closely connected modules in PPI to predict the interaction between proteins encoded by DEGs. Finally, we screened the most critical genes.

### GSEA Enrichment Analysis of Hub Genes Pathway and Biological Function

Gene set enrichment analysis (GSEA; Broad Institute, Inc., Massachusetts Institute of Technology, and Regents of the University of California) is a widely used computational method that determines whether there is a statistically significant difference between two gene groups (20). In this study, we used the GSEA software (version 3.0) to analyze the GO and KEGG pathways of the hub genes.

### Immune State Was Evaluated Based on CIBERSORT and ssGSEA Algorithms

Our study used the CIBERSORT (21) and ssGSEA algorithms (20) to evaluate the immune status of early DKD tissues. With “CIBERSORT” (R packet), we used the CIBERSORT algorithm to analyze gene expression data. Using the standard *P* value <0.05, we screened samples and calculated the percentage of 22 immune cells. Our study compared standardized data with gene sets by using “GSVA” (R package). The ssGSEA algorithm classified genes with common biological functions, chromosomal localization, and physiological regulation. Ultimately, we identified 29 immune-related genes.

### Immunohistochemistry

This study was approved by the Beijing Hospital Institutional Review Board. The ethics approval letter number is 2022BJYYEC-025-01. As described under the approved protocols, DKD kidney biopsy samples from patients were collected by ultrasound-guided renal biopsy. Early DKD patients were defined as having a urine albumin–creatine ratio of between 30 and 300 mg/g and an estimated glomerular filtration rate of >90 ml/min/1.73 m^2^. Biopsy samples included five cases of early DKD and three normal tissues adjacent to the tumor nephrectomy samples. Histological analysis of all patients was performed by investigators who were blinded to the experimental design. Using specific primary antibodies and biotinylated secondary antibodies, immunostaining was performed on five samples of early DKD and three normal samples from nephrectomies. The sections were incubated with rabbit anti-c-Jun (ab40766; antibody diluted 1:100; Abcam, USA), anti-c-EGR1 (ab194357; antibody diluted 1:100; Abcam, USA), anti-c-Fos (ab222699; antibody diluted 1:100; Abcam, USA), and anti-c-ATF3 (ab254268; antibody diluted 1:100; Abcam, USA), followed by secondary antibodies (Cell Signaling Technology, USA). The slides were photographed under a microscope with a digital camera (Nikon Eclipse ci). The imaging system was a Nikon Digital Sight DS-FI2. We used ImageJ software to assess 5–10 horizons (200× magnification) for semiquantitative analysis.

### Statistical Analysis

The analysis in this study was performed using R software version 3.6.2, in which the “limma” package was used for differential gene acquisition, the “cluster Profiler” and “org.Hs.eg.db” packages were used for functional enrichment analysis, and the “survival” package was used to perform Kaplan–Meier survival analysis. The study conducted a statistical analysis between two independent samples based on SPSS Statistics 22 using the *t*-test, and *P <*0.05 was considered statistically significant.

## Results

### Screening of DEGs

**Figure 1:**
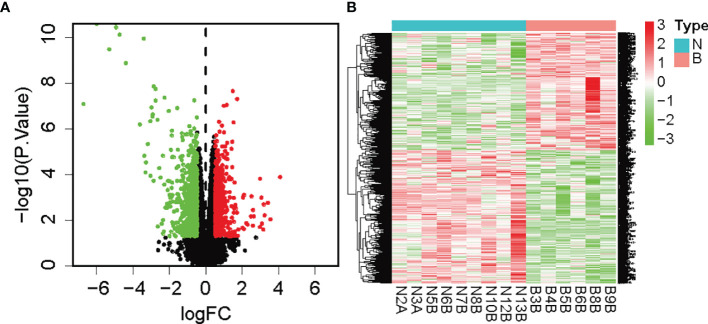
**(A)** Volcano map of DEGs (green was the downregulated gene, red was the upregulated gene, and black was the undifferentiated gene); **(B)** Heatmap of DEGs (note: green was downregulated and red was upregulated).

We analyzed the differences in gene types and visualized genetic variations. Volcano plots were used for figure analysis of identified DEGs. Compared with the normal control, red dots represent the upregulation of genes in the tissues of early DKD patients, while green dots represent the downregulation of genes, as shown in **Figure 1A**. In the heatmap, the limma package of the R language screened 267 DEGs. Compared with the normal control, the red area in early DKD patient tissues represents adequately expressed genes, while the green area represents poorly expressed genes. As shown in **Figure 1B**, 91 genes were adequately expressed and 176 were poorly expressed (see the attached table DEGs 1).

### Weighted Gene Co-Expression Network Analysis (WGCNA)

**Figure 2:**
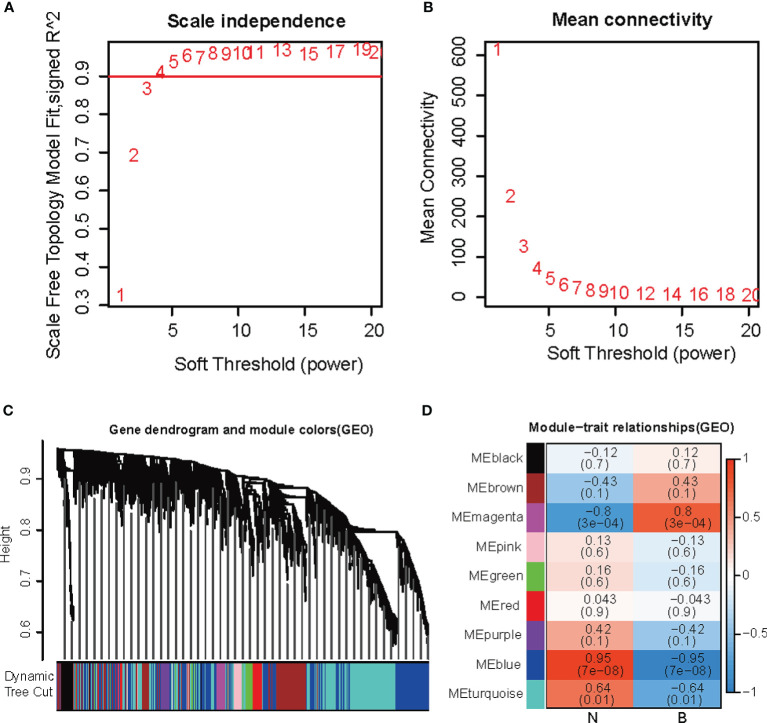
WGCNA co-expression network analysis **(A)** Escale-free fitting index. **(B)** Mean connectivity. **(C)** Each branch in the tree represented a gene, and the color of each module was on behalf of a co-expression module. **(D)** The heatmap for the correlation between the epigenome and early DKD formation traits.

Using WGCNA, we identified the key modules relevant to early DKD formation in the GEO dataset. In **Figures 2A, B**, we analyzed the scale-free fitting index (left) and average connectivity (right) of various soft threshold powers based on the scale-free R2. In **Figure 2C**, the tree graph of all genes is based on different metric (1 − Tom) clustering. Each branch in the tree represents a gene, and the color of each module represents a co-expression module. The heatmap representing the correlation between the epigenome and early DKD formation traits, with each group containing a correlation coefficient and a *P*-value, is shown in **Figure 2D**. Numbers in parentheses on the left represent the number of genes in the corresponding epigenetic module. As shown in **Figure 2**, the most positively correlated gene was magenta, whereas the most negatively correlated gene was blue (see the attached table).

### Venn Diagram

**Figure 3:**
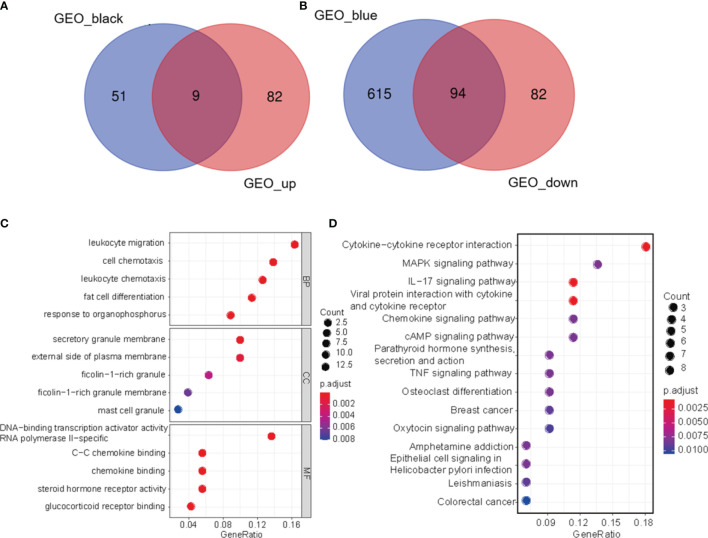
Venn diagram. **(A)** The intersection of up-regulated genes and positively relevant genes in early DKD. **(B)** the intersection of downregulated genes and negatively relevant genes in early DKD. **(C)** Enrichment analysis of GO biological function of DEGs. **(D)** KEGG signaling pathway analysis of DEGs.

Using the Venn Diagram R package (22), we conducted Venn diagram analysis of DEGs and epigenetics filtered from the data set to screen for genes associated with early DKD. These diagrams show overlapping genes and biological complementarities. **Figure 3A** shows the intersection of upregulated and positively relevant genes in early DKD (see the attached table). **Figure 3B** shows the intersection of downregulated and negatively relevant genes in early DKD (see the attached table).

### GO Enrichment Analysis and KEGG Pathway Analysis of Differential Genes

Using the DAVID database, we conducted a GO biological function enrichment analysis for 103 significant differences. In terms of biological processes, differential genes were mainly involved in biological processes, which included leukocyte migration, cell chemotaxis, leukocyte chemotaxis, adipocyte differentiation, and organophosphorus response. In terms of cell composition, the different genes were most abundant in the areas of secretory granules and on the external side of the plasma membrane. In terms of molecular functions, the differential genes were mainly enriched in DNA-binding transcription activator activity, RNA polymerase II-specific, C–C chemokine binding, and chemokine binding, as shown in **Figure 3C**. As shown in **Figure 3D**, KEGG signal pathway enrichment analysis suggested that DEGs were involved in C–C receptor interaction, the IL-17 signaling pathway, and viral protein interaction with cytokines and cytokine receptors. The results of GO functional biological function enrichment analysis and KEGG signaling pathway enrichment analysis indicated that the biological functions relevant to early DKD were immune inflammatory responses and cytokine effects.

### Screening of Hub Genes of Protein Interaction Network

**Figure 4:**
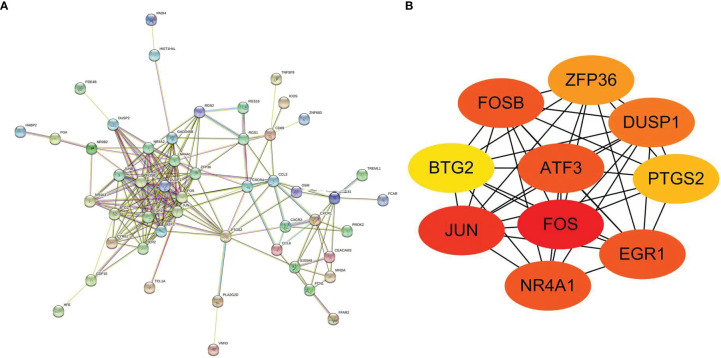
**(A)** Protein–protein interaction network of DEGs. **(B)** Protein–protein interaction network of differentially expressed hub genes (darker color indicated higher grades).

To screen the differential genes that were strongly linked to early DKD, we performed protein interaction network analysis on the basis of 103 differential genes using the STRING database and Cytoscape software, as shown in **Figure 4A**. As a basis, the top 10 positioning hub genes were further screened, and included FOS, JUN, EGR1, ATF3, FOSB, ZFP36, DUSP1, PTGS2, BTG2, and NR4A1, shown in **Figure 4B**. JUN, EGR1, FOS, and ATF3 proteins are closely correlated with other proteins, with darker colors indicating higher grades.

### GSEA Enrichment Analysis of Biological Functions and Pathways of Hub Genes

**Figure 5:**
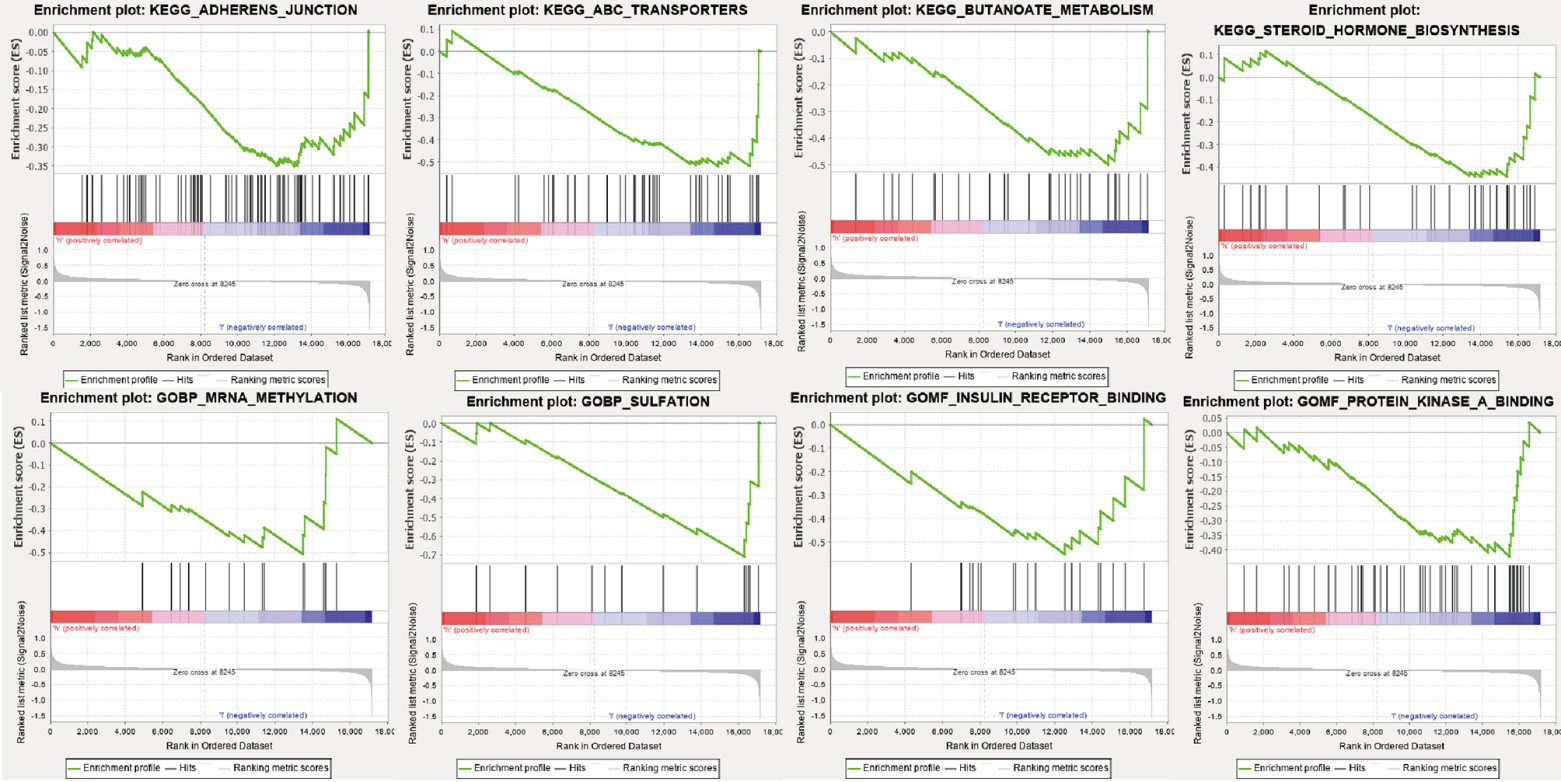
Activated pathways and activated biological functions of GSEA enrichment analysis in the FOS group with low expression.

We found that FOS was a hub gene. We conducted a GSEA enrichment analysis of genes with low expression in the FOS group. As shown in **Figure 5**, four activated biological functions, namely, mRNA methylation, sulfation, insulin receptor binding, and protein kinase A binding, were identified. Also shown in **Figure 5**, are four activated pathways, namely, adherens junction, ABC transporters, butanoate metabolism, and steroid hormone biosynthesis, that were identified.

### Immune Status of DKD Tissues Were Evaluated Based on CIBERSORT and ssGSEA Algorithms

**Figure 6:**
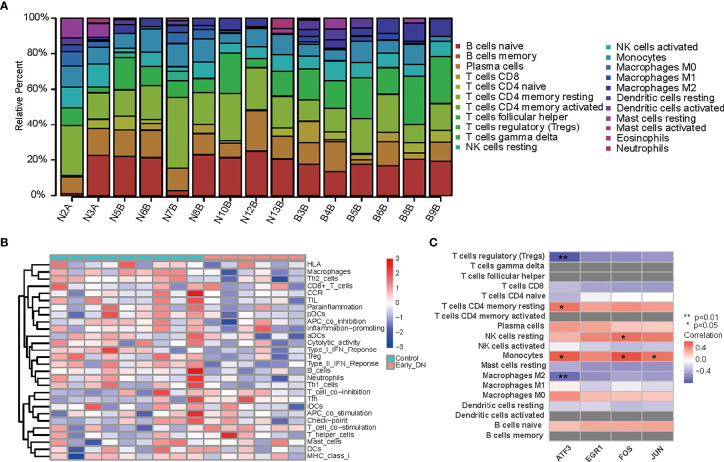
**(A)** Heatmap of 29 immune genes in normal and early DKD tissues based on ssGSEA algorithm. **(B)** Heatmap of the expression levels of 22 kinds of immune cells in normal and early DKD tissues based on the CIBERSORT algorithm. **(C)** Heatmap of hub genes with relevant immune cells infiltration in early DKD tissue.

The heatmap represents the levels of 29 immune genes in normal tissues and early DKD tissues based on the ssGSEA algorithm, as shown in **Figure 6A**. The expression levels of 22 immune cells in normal and early DKD tissues were determined using the CIBERSORT algorithm, as shown in **Figure 6B**. The four hub genes, FOS, EGR1, ATF3, and JUN, were positively correlated with immune cell infiltration in early DKD tissues. ATF3 was positively correlated with monocyte infiltration (*P <*0.05) and memory resting CD4 T cells (*P <*0.05). ATF3 was negatively correlated with M2 macrophages (*P <*0.01) and regulatory T cells (Tregs) (*P <*0.01). The FOS group was positively correlated with the infiltration of monocytes (*P <*0.05) and resting natural killer cells (*P <*0.05). As shown in **Figure 6C**, JUN was positively correlated with monocyte infiltration (*P <*0.05).

### Immunostaining Results

**Figure 7:**
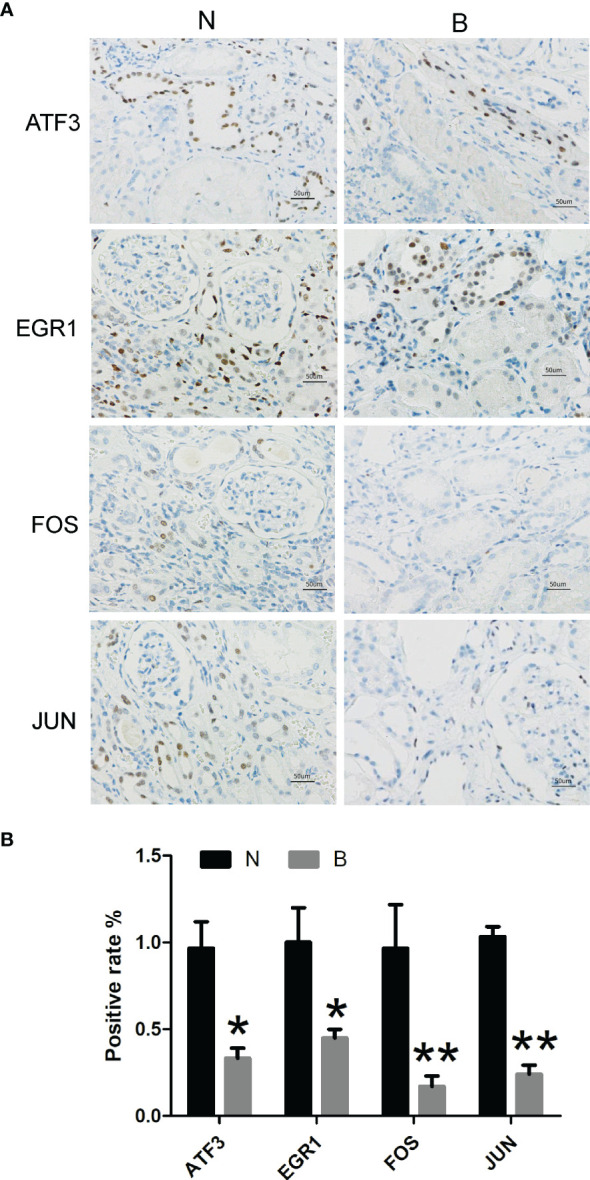
Immunostaining of early DKD and normal samples from the Beijing Hospital to label proteins such as FOS, EGR1, ATF3, and JUN (N, normal kidney tissue; B, early diabetic kidney disease; *P <0.05, **P <0.01).

As shown in **Figure 7**, we performed immunostaining on five early DKD samples and three normal samples from the Beijing Hospital to label proteins such as FOS, EGR1, ATF3, and JUN. The results demonstrated that the relative expression levels of FOS, EGR1, ATF3, and JUN proteins were significantly downregulated in the early DKD group compared with the normal group.

## Discussion

DKD is a common complication of type 2 diabetes, with a high prevalence of 20–50% in patients with diabetes (1). DKD accounts for 44.5% of end-stage renal disease cases in developed countries (23). In line with the global diabetes pandemic, the absolute number of DKD patients is increasing (24). The cost of diabetes management was estimated to be approximately $760 billion in 2019, and it is expected to rise to $845 billion by 2045, with the majority of the costs used to treat related complications (25). Despite the enormous economic health pressure, we urgently require promising clinical biomarkers of early DKD to effectively slow down and, ideally, halt the progression of DKD.

With the rapid development of high-throughput sequencing technology and gene chip technology, deep mining of sequencing data or gene chips in bioinformatics enables extensive and in-depth analysis of the mRNA expression profile of the whole genome. In this study, we explored the relevant target genes and gene interactions that influence the progression of early DKD. As far as we are aware, this is the first study to screen and identify hub genes in early DKD patients and control normal kidney tissue using WGCNA. We validated our conclusions using kidney specimens from the Beijing Hospital. Our study provides a theoretical basis and promising research proposals for the underlying molecular mechanisms, treatment, and prognosis of early DKD.

In this study, we downloaded the mRNA dataset GSE142025 from the GEO database. A total of 267 DEGs were found in the kidney tissue of patients with early DKD and in normal kidney tissue. We conducted GO biological function enrichment, KEGG signaling pathway enrichment, and protein interaction network analyses. Enrichment analysis suggested that C–C receptor interaction and the IL-17 signaling pathway were essential in early DKD. Using a PPI network and module analysis, we identified FOS, EGR1, ATF3, and JUN as hub sites of protein interaction. Meanwhile, the IHC results revealed that the relative expression levels of FOS, EGR1, ATF3, and JUN were significantly downregulated in the early DKD group compared with normal kidney tissue control.

KEGG signal pathway enrichment analysis suggested that DEGs were mainly enriched in the cytokine receptor interaction pathway, namely, the C–C receptor interaction, the IL-17 signaling pathway, and viral protein interaction with cytokines and cytokine receptors. Kim et al. and Mohamed et al. (26, 27) found that the IL-17 signaling pathway is essential for early DKD and that the application of IL-17A could prevent, treat, and reverse DKD effectively. Cytokine biology tends to be complex. They play various roles by interacting with expressed receptors, triggering signaling pathways and releasing cytokines. In early DKD-based network-centric analyses, the C–C receptor interaction pathway was critical. GSEA enrichment analysis of the FOS group of low-expression genes revealed that the activated biological functions included mRNA methylation, insulin receptor binding, and protein kinase A binding.

Fundamental theories and animal experiments were brought into correspondence with the conclusions of this study. 1) Animal experiments (28, 29) have confirmed that epigenetic regulation of gene expression is important for developing early DKD. In the future, methylation changes promise to predict renal function changes in patients with early DKD (30, 31). 2) Early cellular insulin resistance may directly associate to the onset of early DKD (32). Park et al. and Garner et al. implied that increased insulin receptor signaling protected podocytes from high glucose, insulin, and inflammatory cytokines in the environment (33, 34). 3) Glucose transporter 1-mediated glucose influx, which drove glucose metabolism and ATP production, significantly increased cAMP production and protein kinase A activity in hyperglycemia. Extensive studies (35–37) have demonstrated that the cAMP-protein kinase A pathway plays a crucial role in the epigenetic regulation of profibrotic factors in diabetes. In general, multiple biological functions that are interrelated with low FOS expression are fundamental to the pathogenesis of early DKD.

Several studies have indicated that inflammation is the fundamental pathogenesis of DKD (38, 39). Immune regulation is also associated with disease development and progression (40, 41). Surprisingly, we found that the expression of immune response or inflammatory genes was suppressed in early DKD compared with that in normal kidney tissue. 1) As DKD progressed, the levels of pro-inflammatory monocytes and circulating inflammation increased. Monocytes were recruited to the kidneys and differentiated into macrophages. The recruitment of single cells/macrophages is closely related to the progression of DKD (42, 43). The hub genes, ATF3, FOS, and JUN, were positively correlated with monocytes. However, in our study of early DKD, ATF3, FOS, and JUN expression decreased, indicating fewer monocytes. 2) The imbalance of M1/M2 macrophages leads to persistent inflammation and fibrosis, which ultimately results in renal sclerosis with the release of growth factors (44–48). M2 macrophages can protect cells from damage in the DKD microenvironment (49–51). The hub gene, ATF3, was negatively correlated with M2 macrophage infiltration. However, ATF3 expression was decreased, indicating an increase in macrophage M2 in early DKD in our study. 3) Experimental results suggest that Tregs have potential therapeutic value in improving insulin resistance and slowing organ damage by limiting the pro-inflammatory environment (52–54). ATF3 expression is negatively correlated with Treg infiltration. In contrast, ATF3 expression decreased, whereas Tregs increased in early DKD in our study. This is similar to the finding of Fan et al. (55), who found that the expression of immune response or inflammatory genes was suppressed in early DKD but highly upregulated in advanced DKD.

Expression of c-FOS and c-JUN increased glomerular mesangial cell proliferation, which led to the excess production and accumulation of excreted extracellular matrix. This is an important characteristic of DKD (56). The transcription factor EGR1 is involved in the high glucose-induced proliferation and fibrosis of rat glomerular mesangial cells (57). The stress factor ATF3 is induced in the podocytes of patients with DKD, which increases podocyte apoptosis and injury (58). The four genes that were downregulated at an early stage but are likely to be upregulated with the progression of DKD mostly consist of genes associated with kidney disease pathogenesis, such as those related to immune response and fibrosis. In practice, more data must provide more accurate and reliable conclusions.

Our study has the following limitations. First, we need further validation in cell experiments. Cells were prepared from rats with streptozotocin-induced DKD. Construct plasmids that inhibit the expression of target genes and plasmids that promote the high expression of target genes and then transfer them into early diabetic kidney disease cells. We determined whether the protective effects of the cells were attributed to the upregulation of the expression levels of FOS, EGR1, ATF3, and JUN. This will further confirm our findings, and we are in the process of reversing early DKD by genetic modification. However, further research is required. Second, expression of immune response or inflammatory genes was suppressed in early DKD in our study. This appears to contradict our previous research hypothesis. However, it is a further immune supplement to the mechanism of early DKD. Inflammation is lower in early DKD than in normal kidney tissues, leading to an immune imbalance. Our findings are critical, as they provide a new perspective on the pathogenesis of early DKD. Finally, there are a few early DKD gene chips in the GEO database. We look forward to more genetic data on the differences between early DKD and normal kidney tissue to provide more precise and reliable results.

## Summary

From a bioinformatics perspective, this study revealed early interrelated pathogenic genes. Compared to the normal patient group, the relative expression of FOS, EGR1, ATF3, and JUN proteins in the early DKD group was significantly downregulated. The four hub genes, FOS, EGR1, ATF3, and JUN, were strongly associated with the infiltration of monocytes, macrophage M2, and Tregs. We verified that the expression of immune response or inflammatory genes was suppressed in early DKD. Meanwhile, the FOS group of low-expression genes showed that the activated biological functions included mRNA methylation, insulin receptor binding, and protein kinase A binding. DEGs were mainly enriched in C–C receptor interaction and the IL-17 signaling pathway. Thus, these genes and pathways may be promising therapeutic targets for early DKD.

## Data Availability Statement

The datasets presented in this study can be found in online repositories. The names of the repository/repositories and accession number(s) can be found in the article/**Supplementary Material**.

## Ethics Statement

The studies involving human participants were reviewed and approved by the Beijing Hospital Ethics Committee. Written informed consent for participation was not required for this study in accordance with the national legislation and the institutional requirements.

## Author Contributions

RW consulted literatures and wrote the manuscript. JQ assisted with writing and revising the manuscript. DC performed immunostaining of kidney issue. QP and LG designed the study. All authors listed have made a substantial, direct, and intellectual contribution to the work and approved it for publication.

## Funding

This work was supported by the National Natural Science Foundation of China (82170848) and the Beijing Hospital Project (BJ-2021-200).

## Conflict of Interest

The authors declare that the research was conducted in the absence of any commercial or financial relationships that could be construed as a potential conflict of interest.

## Publisher’s Note

All claims expressed in this article are solely those of the authors and do not necessarily represent those of their affiliated organizations, or those of the publisher, the editors and the reviewers. Any product that may be evaluated in this article, or claim that may be made by its manufacturer, is not guaranteed or endorsed by the publisher.

## Abbreviations

DKD, Diabetic Kidney Disease; IHC, immunohistochemistry; DEGs, Differentially Expressed Genes; NCBI, National Center for Biotechnology Information; GEO, Gene Expression Omnibus; GO, Gene Ontology; MF, Molecular function; CC, Cellular components; BP, Biological Process; KEGG, Kyoto Encyclopedia of Genes and Genomes; PPI, Protein–Protein Interaction; GSEA, Gene Set Enrichment Analysis; Tregs, T cells regulatory.
