# Corrigendum: Screening and identification of hub genes in the development of early diabetic kidney disease based on weighted gene co-expression network analysis

**DOI:** 10.3389/fendo.2024.1367390

**Published:** 2024-01-19

**Authors:** Ran Wei, Jingtao Qiao, Di Cui, Qi Pan, Lixin Guo

**Affiliations:** ^1^ Department of Endocrinology, Peking University Fifth School of Clinical Medicine, Beijing, China; ^2^ Department of Endocrinology, Beijing Hospital, National Center of Gerontology, Institute of Geriatric Medicine, Chinese Academy of Medical Sciences, Beijing, China; ^3^ Department of Pathology, Beijing Hospital, National Center of Gerontology, Institute of Geriatric Medicine, Chinese Academy of Medical Sciences, Beijing, China

**Keywords:** bioinformatics, WGCNA, GSEA analysis, early diabetic kidney disease, hub gene

In the published article, there was an error in affiliation 1. Instead of “Department of Endocrinology, Beijing Hospital, National Center of Gerontology, Institute of Geriatric Medicine, Chinese Academy of Medical Sciences, Beijing, China”, it should be “Department of Endocrinology, Peking University Fifth School of Clinical Medicine, Beijing, China”.

In the published article, there was an error in affiliation 2. Instead of “Department of Endocrinology, Peking University Fifth School of Clinical Medicine, Beijing, China”, it should be “Department of Endocrinology, Beijing Hospital, National Center of Gerontology, Institute of Geriatric Medicine, Chinese Academy of Medical Sciences, Beijing, China”.

In the published article, there was an error regarding the affiliations for Ran Wei and Lixin Guo. Instead of having affiliations 1 and 2, they should only have *1 Department of Endocrinology, Peking University Fifth School of Clinical Medicine, Beijing, China*.

In the published article, there was an error regarding the affiliation for Jingtao Qiao. Instead of having affiliation 1, they should have *2 Department of Endocrinology, Beijing Hospital, National Center of Gerontology, Institute of Geriatric Medicine, Chinese Academy of Medical Sciences, Beijing, China*.

The authors apologize for these errors and state that these do not change the scientific conclusions of the article in any way. The original article has been updated.

