# Supplementary figures and images for: Screening and Identification of Hub Genes in the Development of Early Diabetic Kidney Disease Based on Weighted Gene Co-Expression Network Analysis
Source: Front Endocrinol (Lausanne). 2022 Jun 3;13:883658. doi: 10.3389/fendo.2022.883658 (PMC9204256; doi:10.3389/fendo.2022.883658)

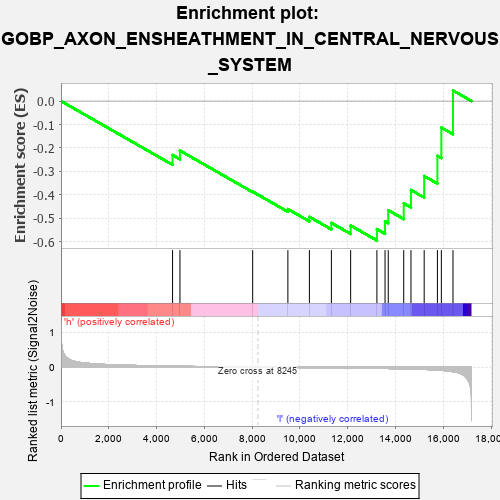

Supplement: Supplementary file 1 [file DataSheet_1.zip › enplot_GOBP_AXON_ENSHEATHMENT_IN_CENTRAL_NERVOUS_SYSTEM_336.png]

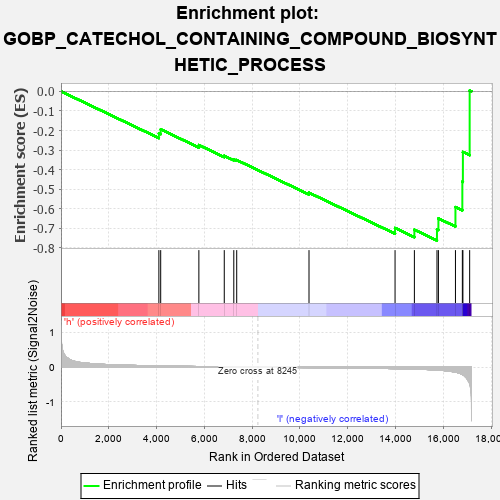

Supplement: Supplementary file 1 [file DataSheet_1.zip › enplot_GOBP_CATECHOL_CONTAINING_COMPOUND_BIOSYNTHETIC_PROCESS_504.png]

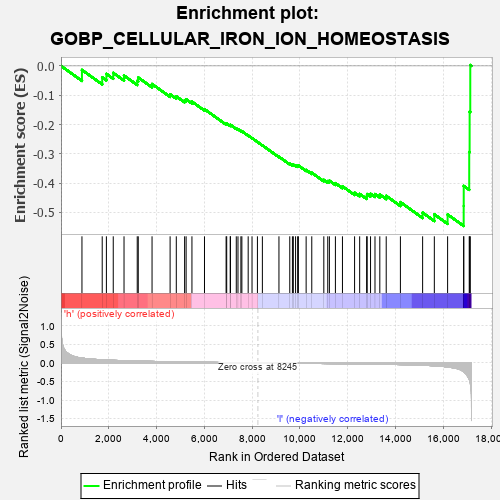

Supplement: Supplementary file 1 [file DataSheet_1.zip › enplot_GOBP_CELLULAR_IRON_ION_HOMEOSTASIS_570.png]

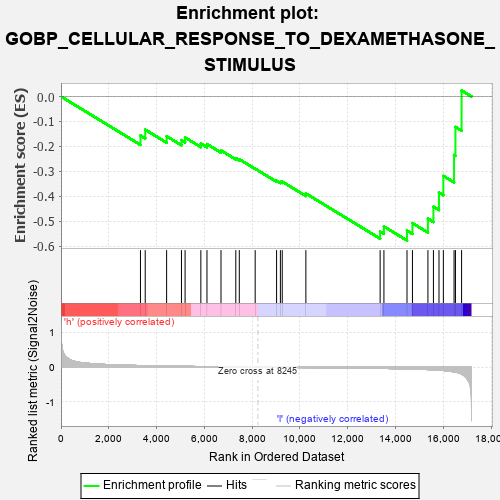

Supplement: Supplementary file 1 [file DataSheet_1.zip › enplot_GOBP_CELLULAR_RESPONSE_TO_DEXAMETHASONE_STIMULUS_390.png]

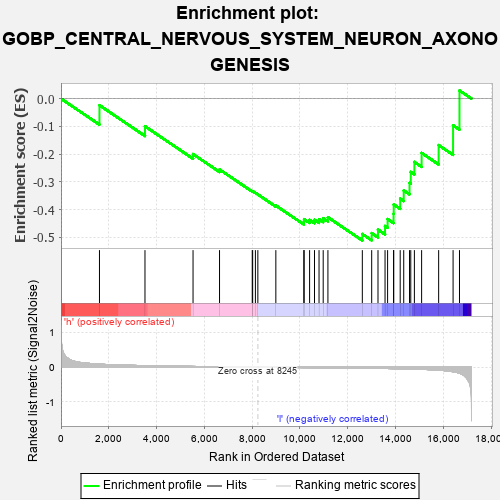

Supplement: Supplementary file 1 [file DataSheet_1.zip › enplot_GOBP_CENTRAL_NERVOUS_SYSTEM_NEURON_AXONOGENESIS_501.png]

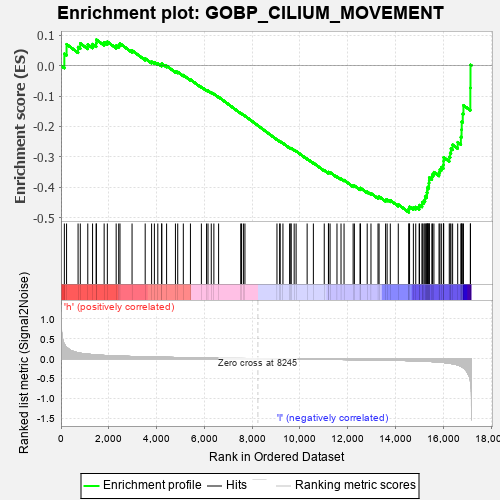

Supplement: Supplementary file 1 [file DataSheet_1.zip › enplot_GOBP_CILIUM_MOVEMENT_435.png]

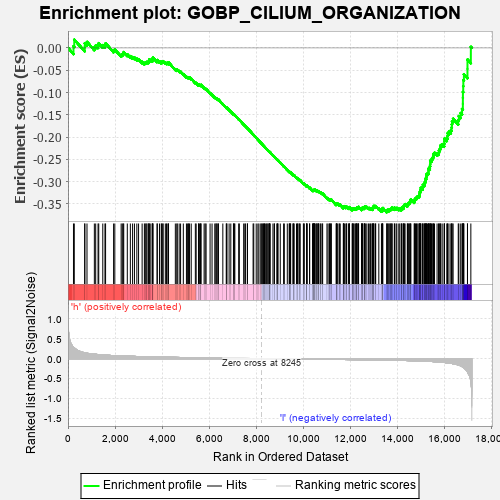

Supplement: Supplementary file 1 [file DataSheet_1.zip › enplot_GOBP_CILIUM_ORGANIZATION_354.png]

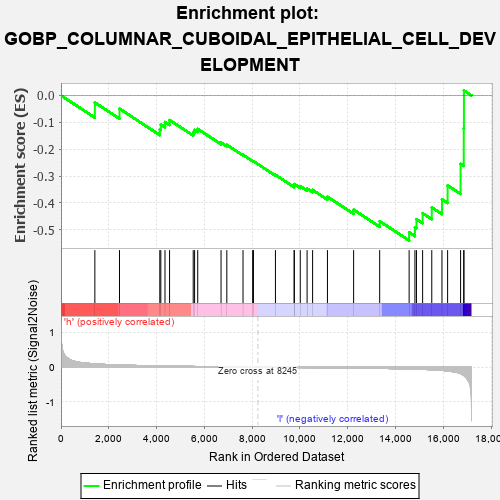

Supplement: Supplementary file 1 [file DataSheet_1.zip › enplot_GOBP_COLUMNAR_CUBOIDAL_EPITHELIAL_CELL_DEVELOPMENT_462.png]

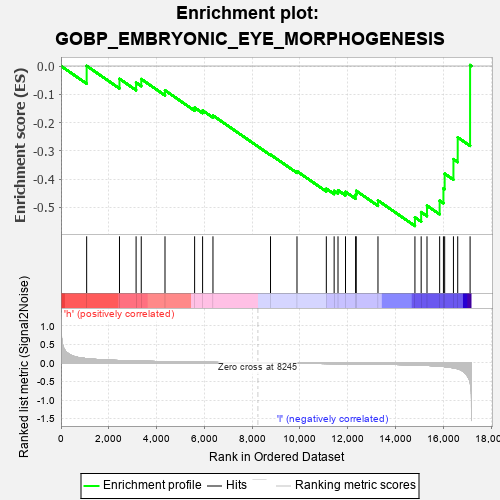

Supplement: Supplementary file 1 [file DataSheet_1.zip › enplot_GOBP_EMBRYONIC_EYE_MORPHOGENESIS_567.png]

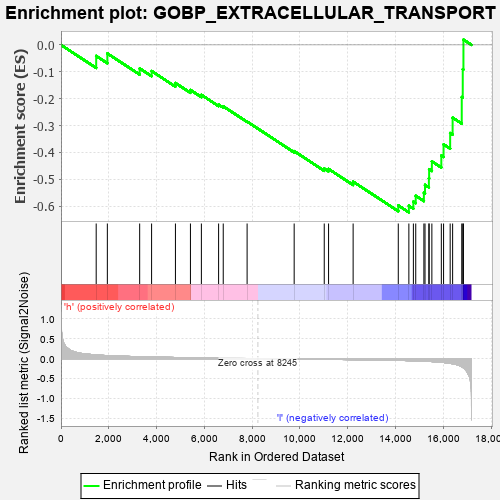

Supplement: Supplementary file 1 [file DataSheet_1.zip › enplot_GOBP_EXTRACELLULAR_TRANSPORT_360.png]

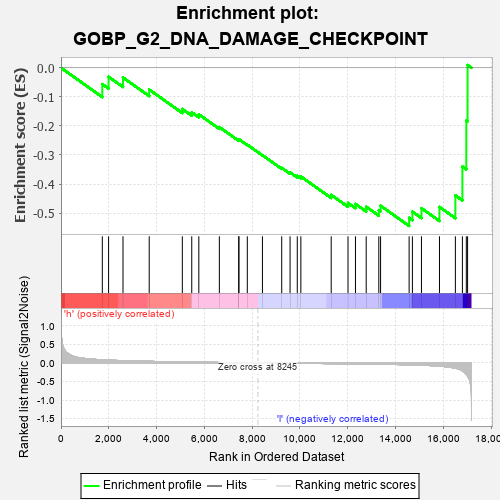

Supplement: Supplementary file 1 [file DataSheet_1.zip › enplot_GOBP_G2_DNA_DAMAGE_CHECKPOINT_582.png]

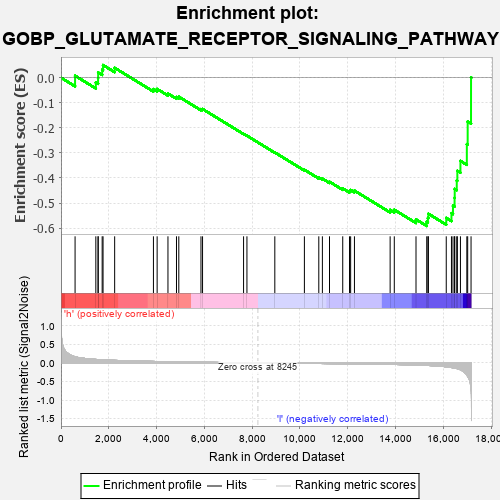

Supplement: Supplementary file 1 [file DataSheet_1.zip › enplot_GOBP_GLUTAMATE_RECEPTOR_SIGNALING_PATHWAY_597.png]

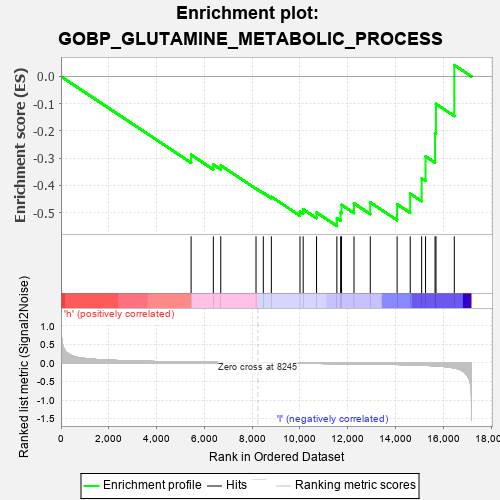

Supplement: Supplementary file 1 [file DataSheet_1.zip › enplot_GOBP_GLUTAMINE_METABOLIC_PROCESS_339.png]

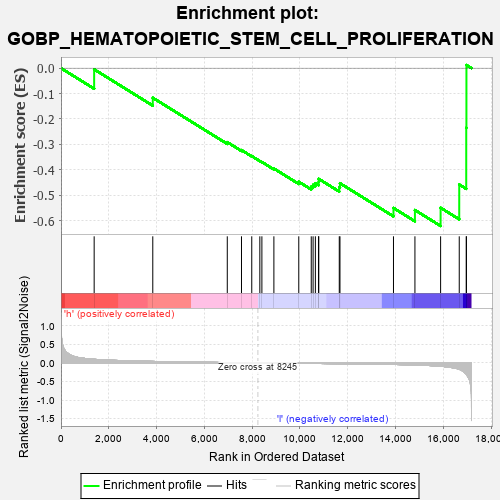

Supplement: Supplementary file 1 [file DataSheet_1.zip › enplot_GOBP_HEMATOPOIETIC_STEM_CELL_PROLIFERATION_471.png]

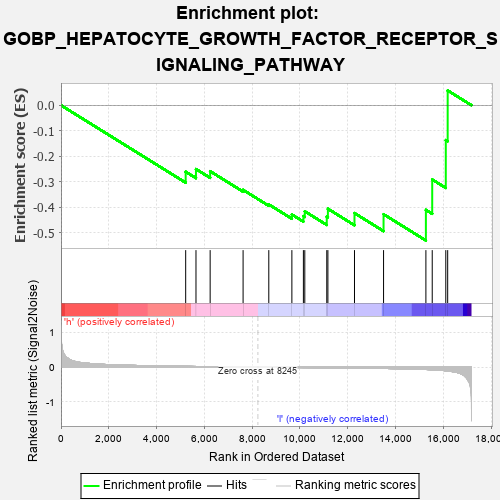

Supplement: Supplementary file 1 [file DataSheet_1.zip › enplot_GOBP_HEPATOCYTE_GROWTH_FACTOR_RECEPTOR_SIGNALING_PATHWAY_549.png]

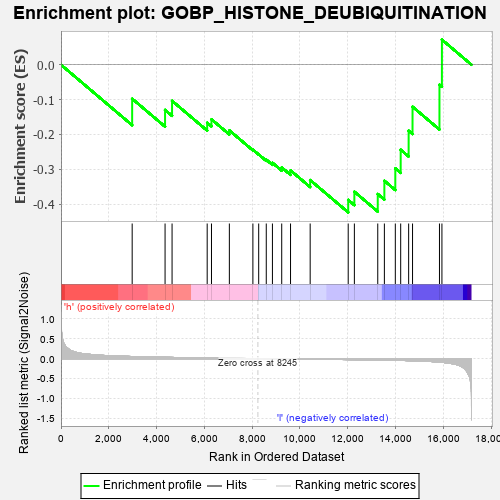

Supplement: Supplementary file 1 [file DataSheet_1.zip › enplot_GOBP_HISTONE_DEUBIQUITINATION_387.png]

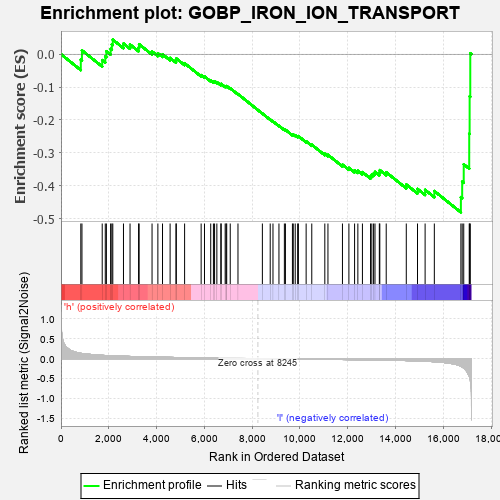

Supplement: Supplementary file 1 [file DataSheet_1.zip › enplot_GOBP_IRON_ION_TRANSPORT_372.png]

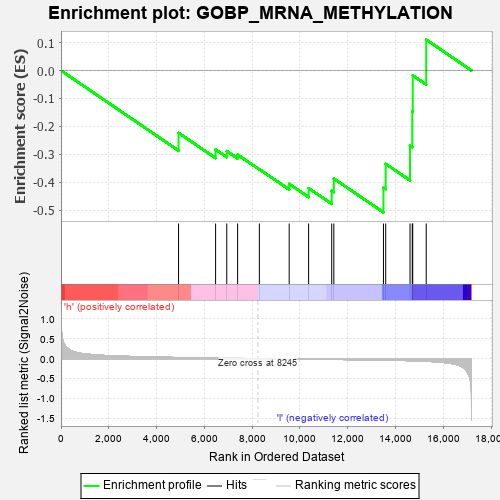

Supplement: Supplementary file 1 [file DataSheet_1.zip › enplot_GOBP_MRNA_METHYLATION_450.png]

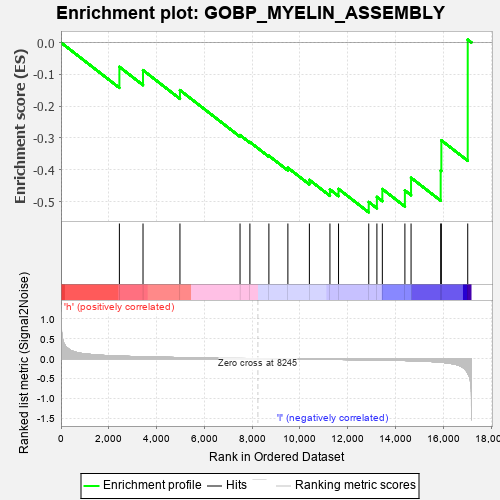

Supplement: Supplementary file 1 [file DataSheet_1.zip › enplot_GOBP_MYELIN_ASSEMBLY_495.png]

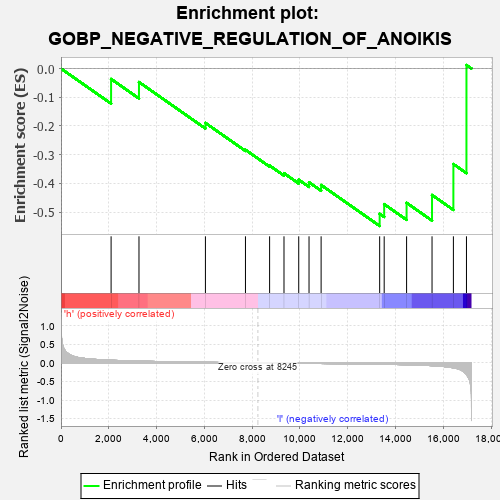

Supplement: Supplementary file 1 [file DataSheet_1.zip › enplot_GOBP_NEGATIVE_REGULATION_OF_ANOIKIS_459.png]

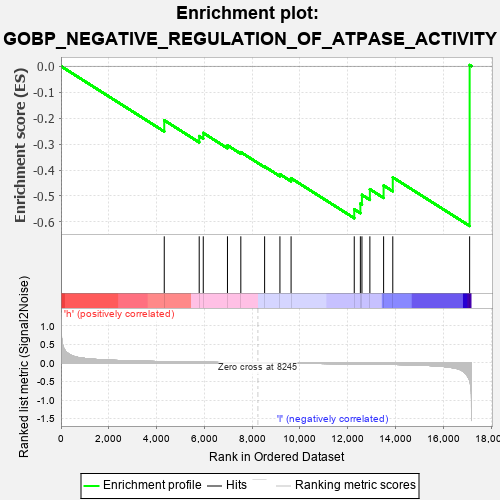

Supplement: Supplementary file 1 [file DataSheet_1.zip › enplot_GOBP_NEGATIVE_REGULATION_OF_ATPASE_ACTIVITY_381.png]

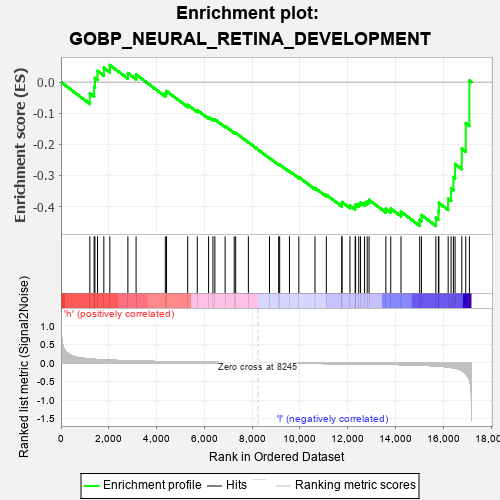

Supplement: Supplementary file 1 [file DataSheet_1.zip › enplot_GOBP_NEURAL_RETINA_DEVELOPMENT_588.png]

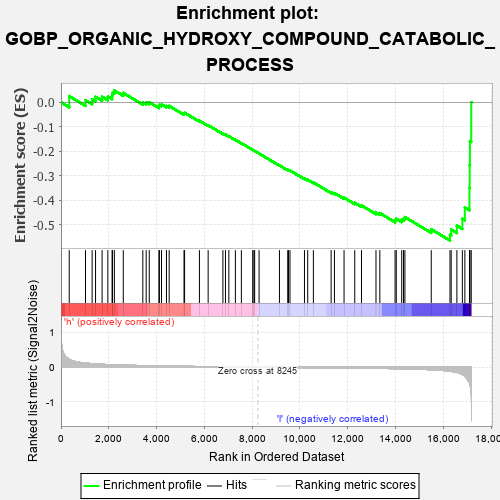

Supplement: Supplementary file 1 [file DataSheet_1.zip › enplot_GOBP_ORGANIC_HYDROXY_COMPOUND_CATABOLIC_PROCESS_357.png]

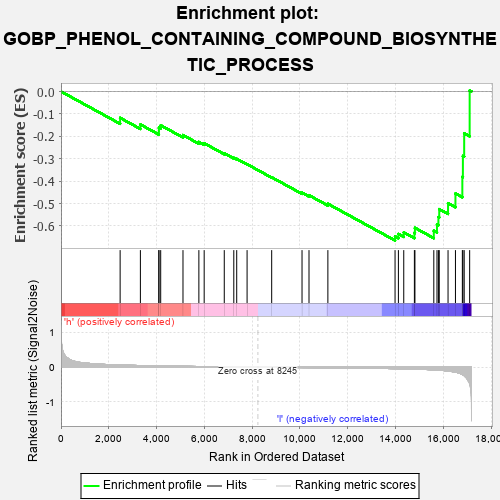

Supplement: Supplementary file 1 [file DataSheet_1.zip › enplot_GOBP_PHENOL_CONTAINING_COMPOUND_BIOSYNTHETIC_PROCESS_363.png]

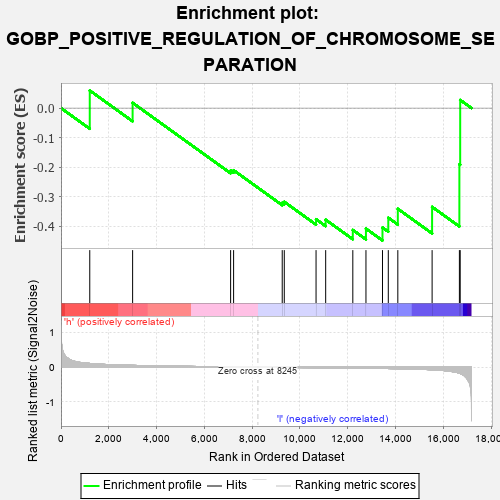

Supplement: Supplementary file 1 [file DataSheet_1.zip › enplot_GOBP_POSITIVE_REGULATION_OF_CHROMOSOME_SEPARATION_579.png]

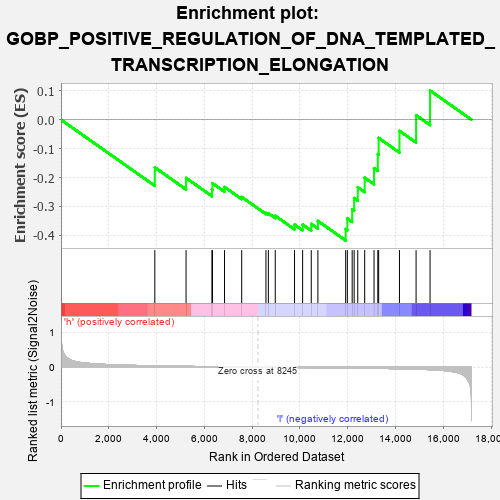

Supplement: Supplementary file 1 [file DataSheet_1.zip › enplot_GOBP_POSITIVE_REGULATION_OF_DNA_TEMPLATED_TRANSCRIPTION_ELONGATION_552.png]

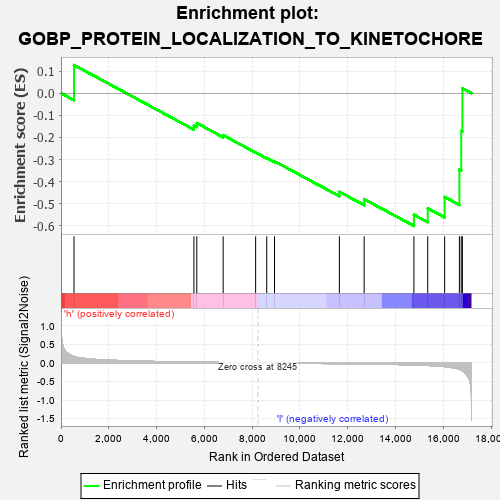

Supplement: Supplementary file 1 [file DataSheet_1.zip › enplot_GOBP_PROTEIN_LOCALIZATION_TO_KINETOCHORE_477.png]

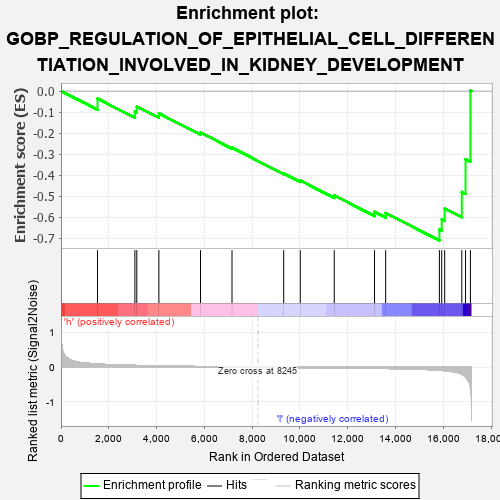

Supplement: Supplementary file 1 [file DataSheet_1.zip › enplot_GOBP_REGULATION_OF_EPITHELIAL_CELL_DIFFERENTIATION_INVOLVED_IN_KIDNEY_DEVELOPMENT_516.png]

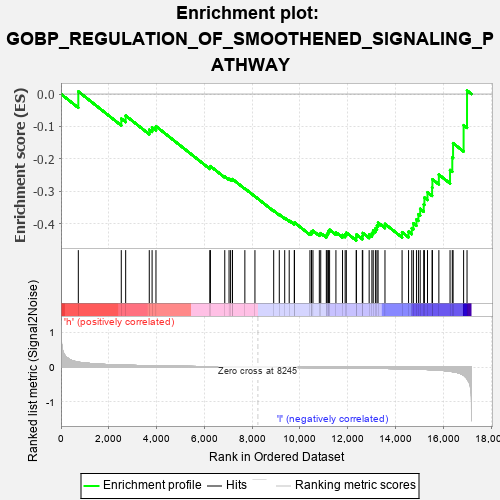

Supplement: Supplementary file 1 [file DataSheet_1.zip › enplot_GOBP_REGULATION_OF_SMOOTHENED_SIGNALING_PATHWAY_384.png]

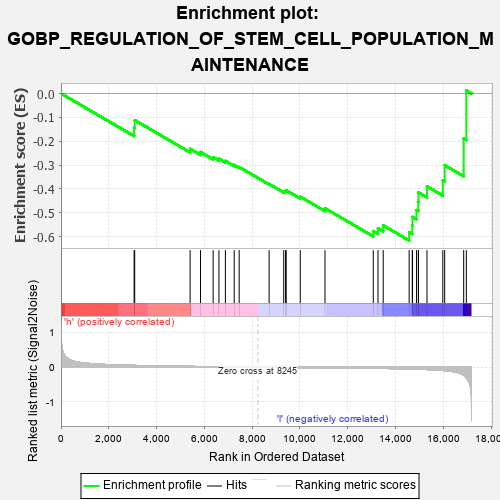

Supplement: Supplementary file 1 [file DataSheet_1.zip › enplot_GOBP_REGULATION_OF_STEM_CELL_POPULATION_MAINTENANCE_513.png]

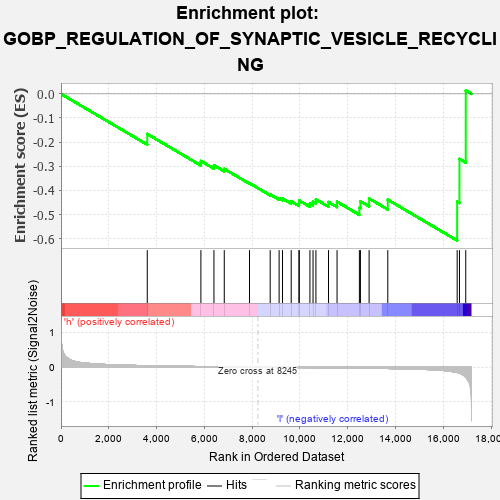

Supplement: Supplementary file 1 [file DataSheet_1.zip › enplot_GOBP_REGULATION_OF_SYNAPTIC_VESICLE_RECYCLING_441.png]

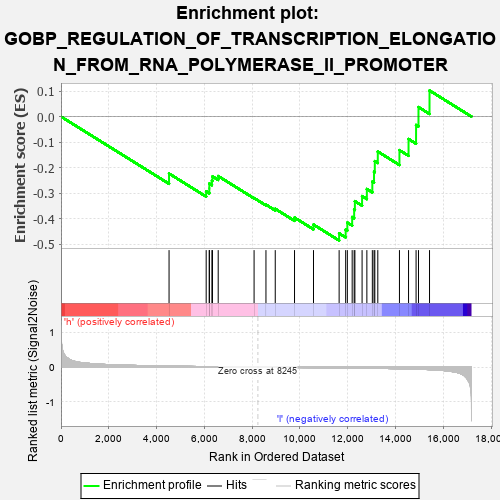

Supplement: Supplementary file 1 [file DataSheet_1.zip › enplot_GOBP_REGULATION_OF_TRANSCRIPTION_ELONGATION_FROM_RNA_POLYMERASE_II_PROMOTER_318.png]

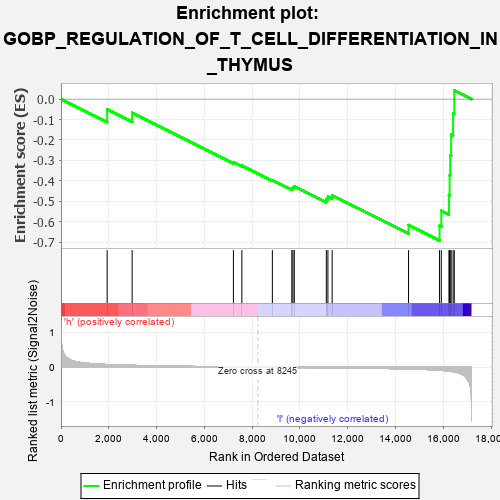

Supplement: Supplementary file 1 [file DataSheet_1.zip › enplot_GOBP_REGULATION_OF_T_CELL_DIFFERENTIATION_IN_THYMUS_348.png]

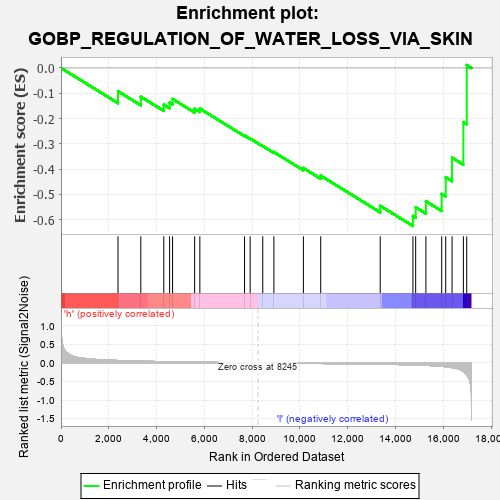

Supplement: Supplementary file 1 [file DataSheet_1.zip › enplot_GOBP_REGULATION_OF_WATER_LOSS_VIA_SKIN_534.png]

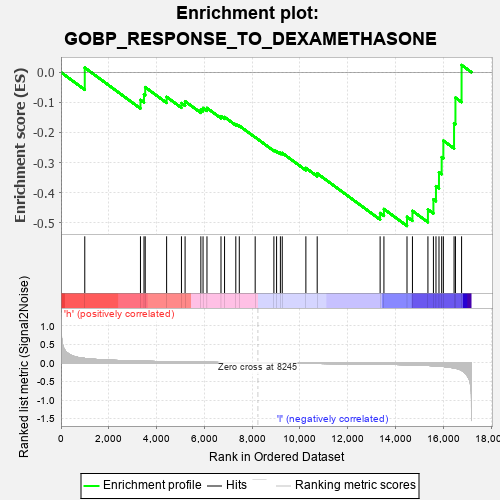

Supplement: Supplementary file 1 [file DataSheet_1.zip › enplot_GOBP_RESPONSE_TO_DEXAMETHASONE_408.png]

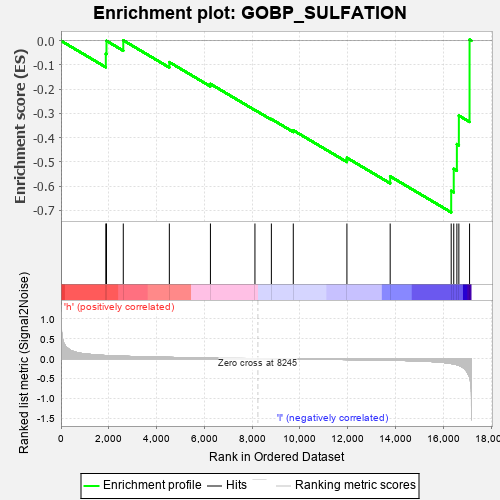

Supplement: Supplementary file 1 [file DataSheet_1.zip › enplot_GOBP_SULFATION_585.png]

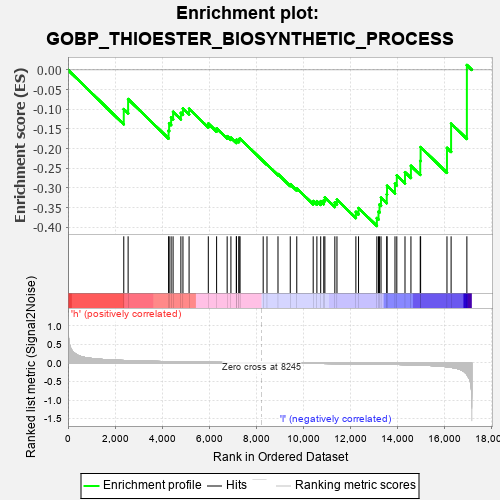

Supplement: Supplementary file 1 [file DataSheet_1.zip › enplot_GOBP_THIOESTER_BIOSYNTHETIC_PROCESS_555.png]

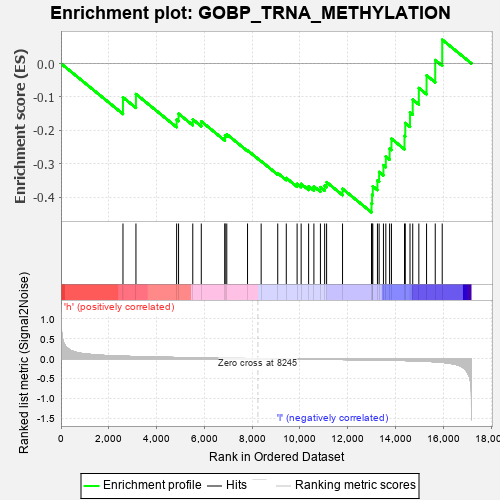

Supplement: Supplementary file 1 [file DataSheet_1.zip › enplot_GOBP_TRNA_METHYLATION_321.png]

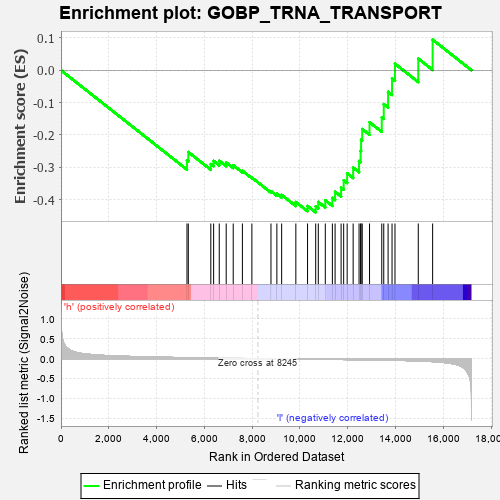

Supplement: Supplementary file 1 [file DataSheet_1.zip › enplot_GOBP_TRNA_TRANSPORT_519.png]

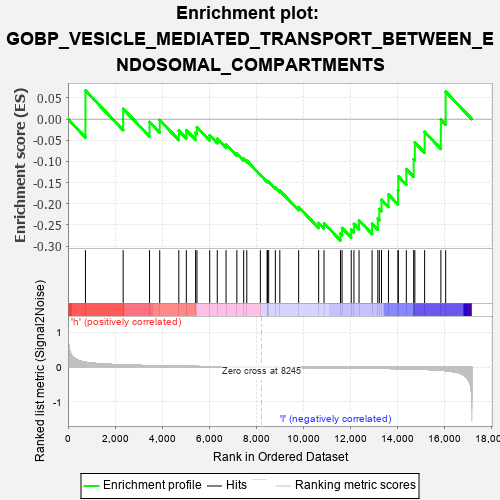

Supplement: Supplementary file 1 [file DataSheet_1.zip › enplot_GOBP_VESICLE_MEDIATED_TRANSPORT_BETWEEN_ENDOSOMAL_COMPARTMENTS_474.png]

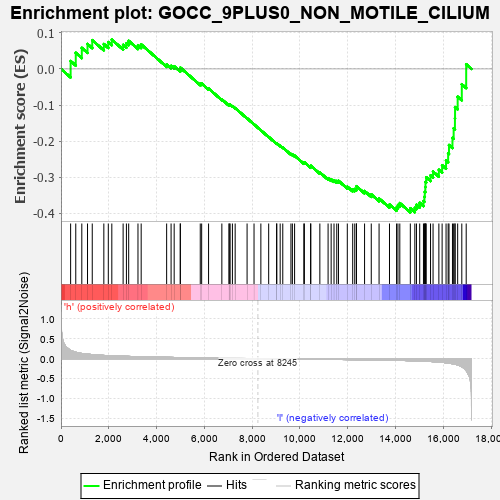

Supplement: Supplementary file 1 [file DataSheet_1.zip › enplot_GOCC_9PLUS0_NON_MOTILE_CILIUM_465.png]

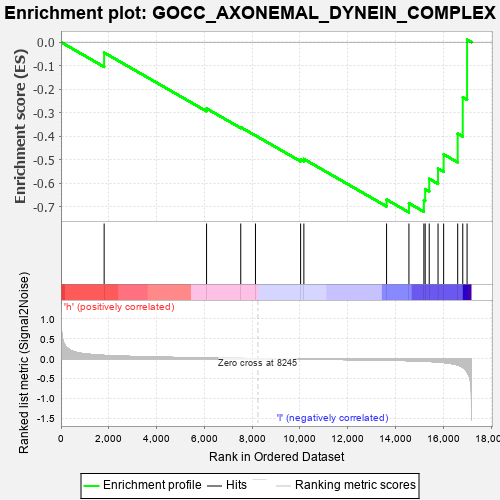

Supplement: Supplementary file 1 [file DataSheet_1.zip › enplot_GOCC_AXONEMAL_DYNEIN_COMPLEX_486.png]

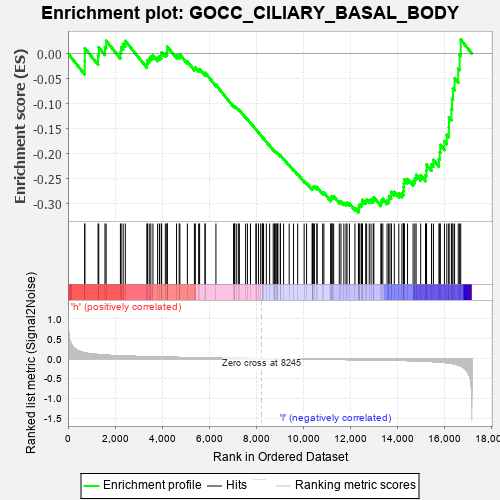

Supplement: Supplementary file 1 [file DataSheet_1.zip › enplot_GOCC_CILIARY_BASAL_BODY_375.png]

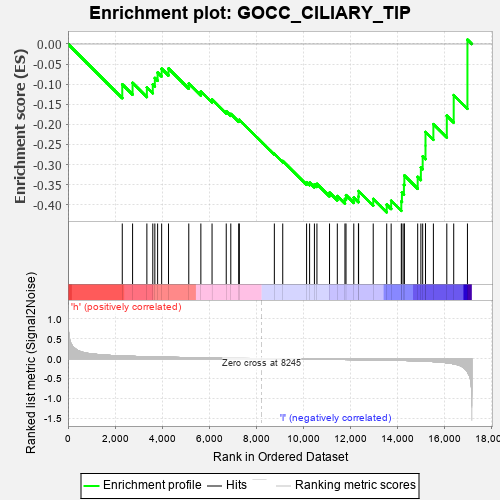

Supplement: Supplementary file 1 [file DataSheet_1.zip › enplot_GOCC_CILIARY_TIP_600.png]

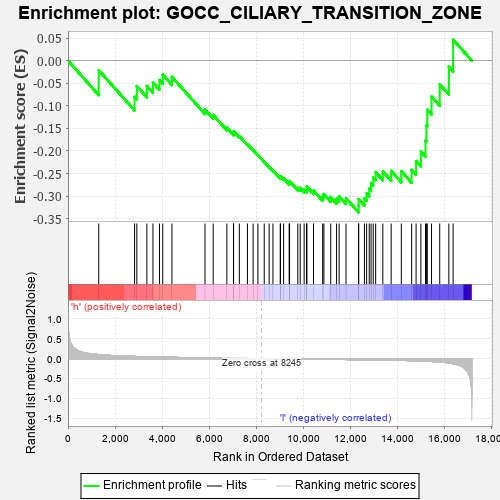

Supplement: Supplementary file 1 [file DataSheet_1.zip › enplot_GOCC_CILIARY_TRANSITION_ZONE_369.png]

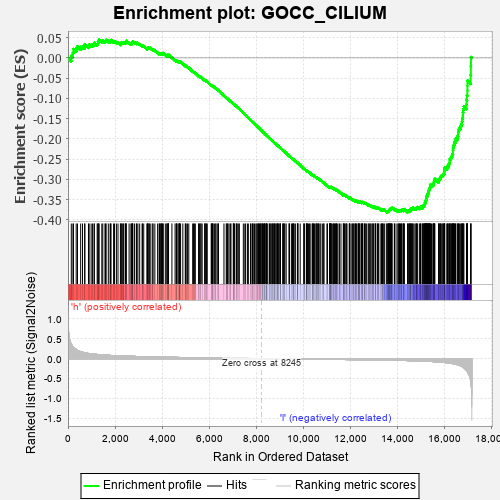

Supplement: Supplementary file 1 [file DataSheet_1.zip › enplot_GOCC_CILIUM_417.png]

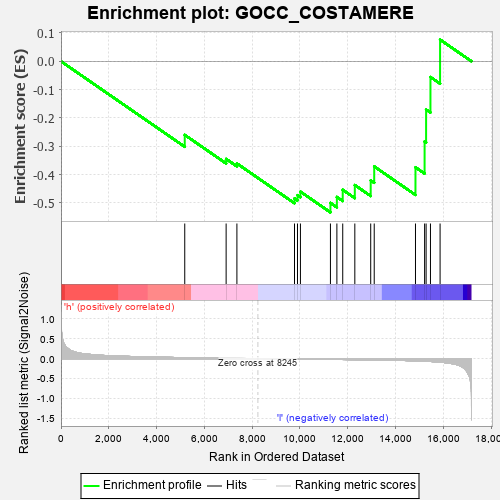

Supplement: Supplementary file 1 [file DataSheet_1.zip › enplot_GOCC_COSTAMERE_309.png]

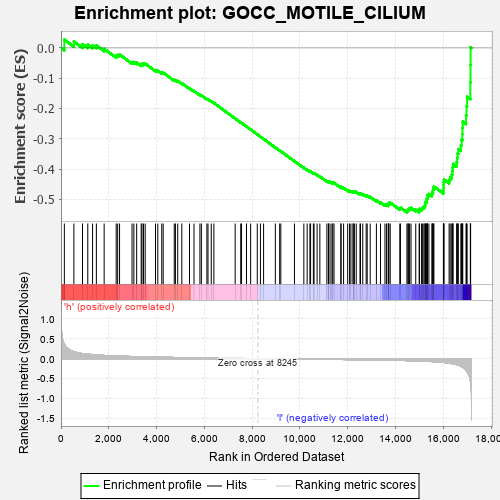

Supplement: Supplementary file 1 [file DataSheet_1.zip › enplot_GOCC_MOTILE_CILIUM_366.png]

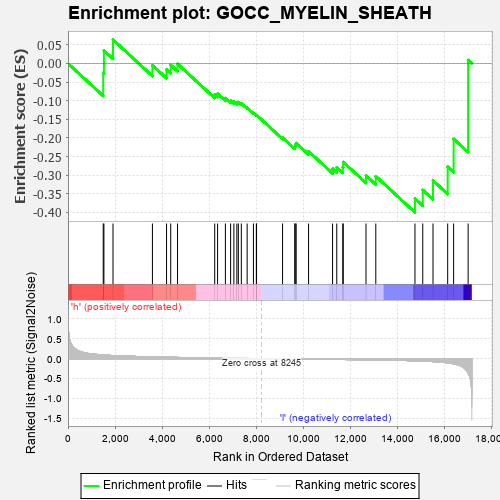

Supplement: Supplementary file 1 [file DataSheet_1.zip › enplot_GOCC_MYELIN_SHEATH_543.png]

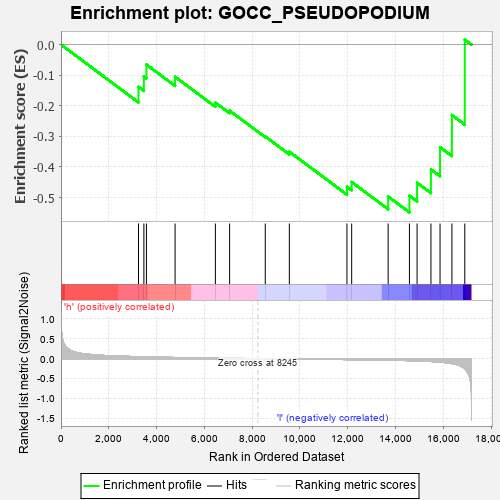

Supplement: Supplementary file 1 [file DataSheet_1.zip › enplot_GOCC_PSEUDOPODIUM_399.png]

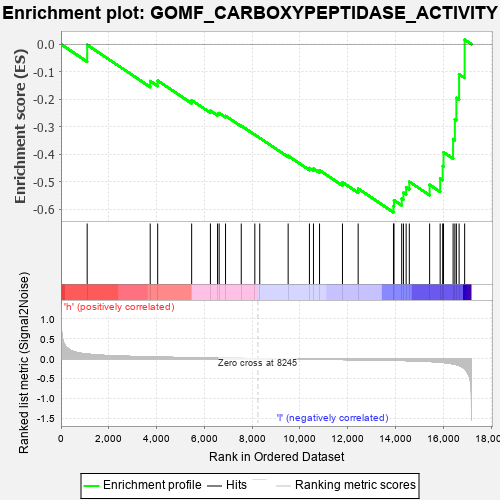

Supplement: Supplementary file 1 [file DataSheet_1.zip › enplot_GOMF_CARBOXYPEPTIDASE_ACTIVITY_453.png]

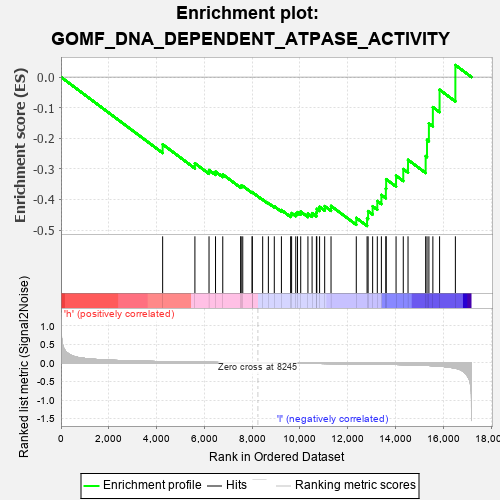

Supplement: Supplementary file 1 [file DataSheet_1.zip › enplot_GOMF_DNA_DEPENDENT_ATPASE_ACTIVITY_315.png]

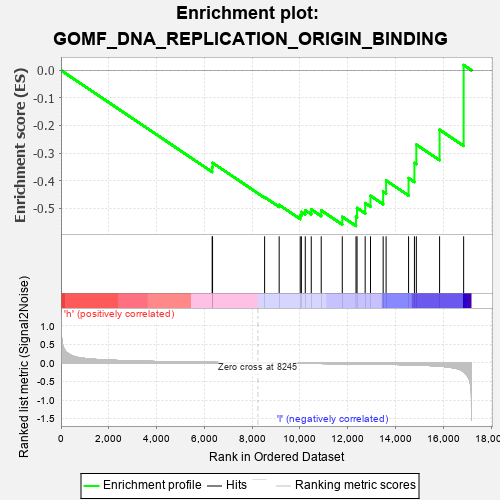

Supplement: Supplementary file 1 [file DataSheet_1.zip › enplot_GOMF_DNA_REPLICATION_ORIGIN_BINDING_420.png]

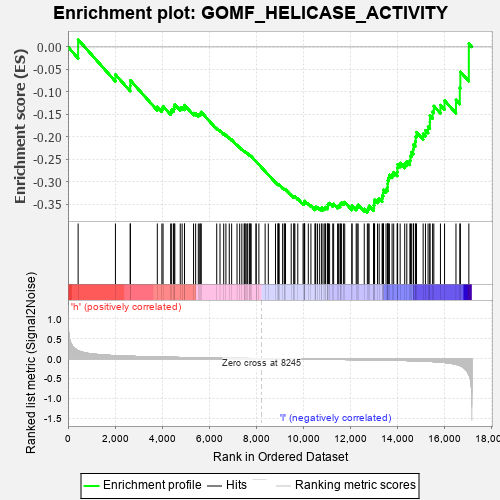

Supplement: Supplementary file 1 [file DataSheet_1.zip › enplot_GOMF_HELICASE_ACTIVITY_414.png]

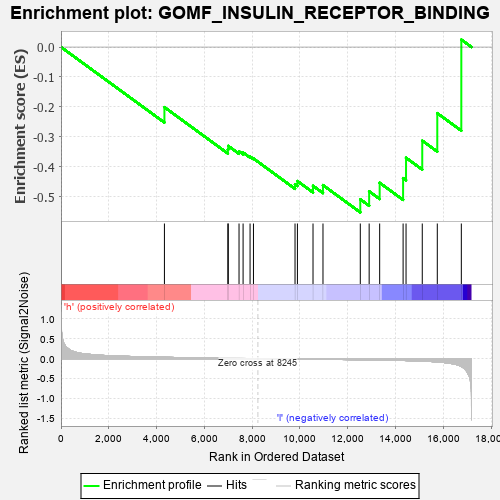

Supplement: Supplementary file 1 [file DataSheet_1.zip › enplot_GOMF_INSULIN_RECEPTOR_BINDING_312.png]

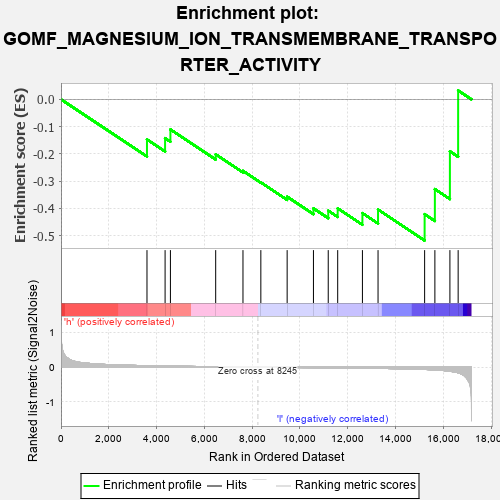

Supplement: Supplementary file 1 [file DataSheet_1.zip › enplot_GOMF_MAGNESIUM_ION_TRANSMEMBRANE_TRANSPORTER_ACTIVITY_594.png]

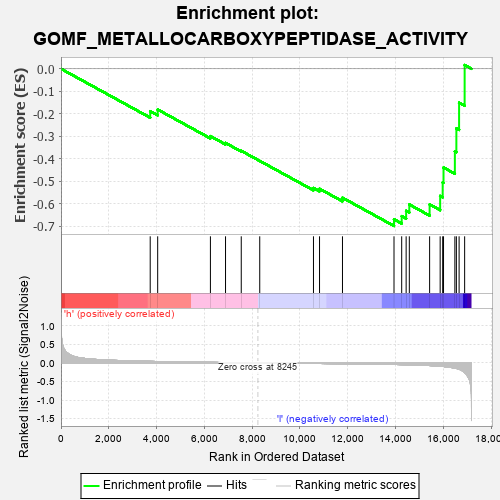

Supplement: Supplementary file 1 [file DataSheet_1.zip › enplot_GOMF_METALLOCARBOXYPEPTIDASE_ACTIVITY_507.png]

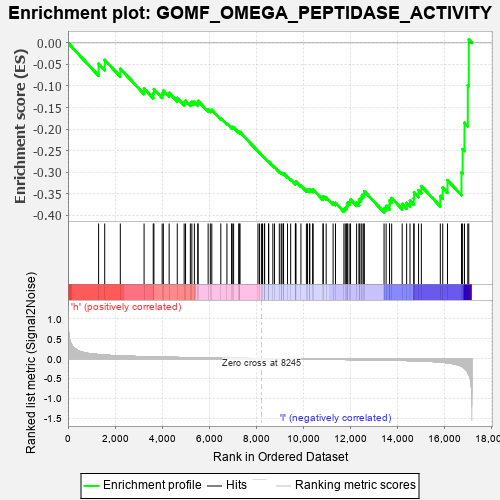

Supplement: Supplementary file 1 [file DataSheet_1.zip › enplot_GOMF_OMEGA_PEPTIDASE_ACTIVITY_342.png]

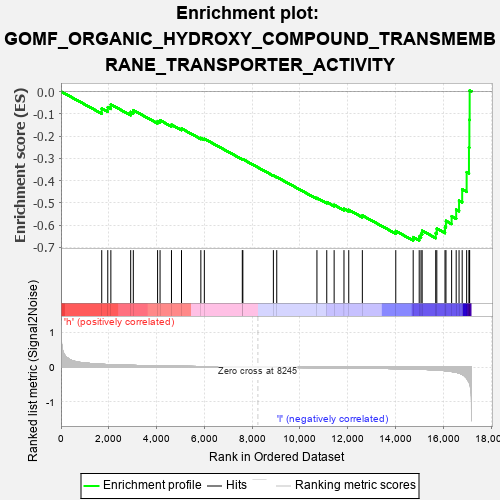

Supplement: Supplementary file 1 [file DataSheet_1.zip › enplot_GOMF_ORGANIC_HYDROXY_COMPOUND_TRANSMEMBRANE_TRANSPORTER_ACTIVITY_429.png]

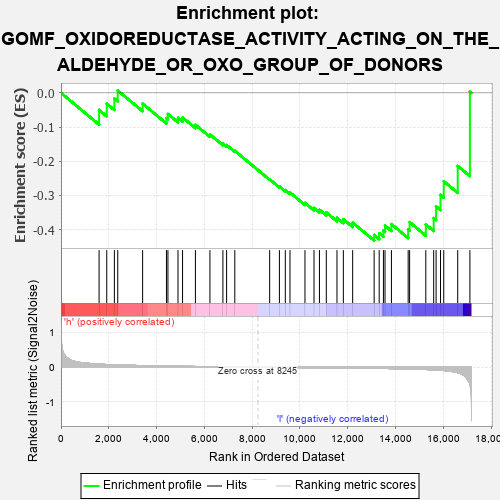

Supplement: Supplementary file 1 [file DataSheet_1.zip › enplot_GOMF_OXIDOREDUCTASE_ACTIVITY_ACTING_ON_THE_ALDEHYDE_OR_OXO_GROUP_OF_DONORS_444.png]

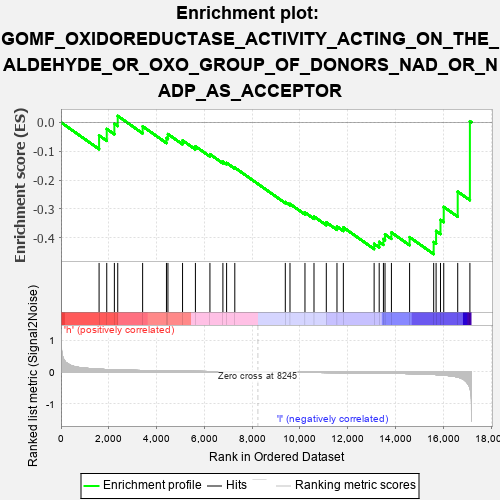

Supplement: Supplementary file 1 [file DataSheet_1.zip › enplot_GOMF_OXIDOREDUCTASE_ACTIVITY_ACTING_ON_THE_ALDEHYDE_OR_OXO_GROUP_OF_DONORS_NAD_OR_NADP_AS_ACCEPTOR_396.png]

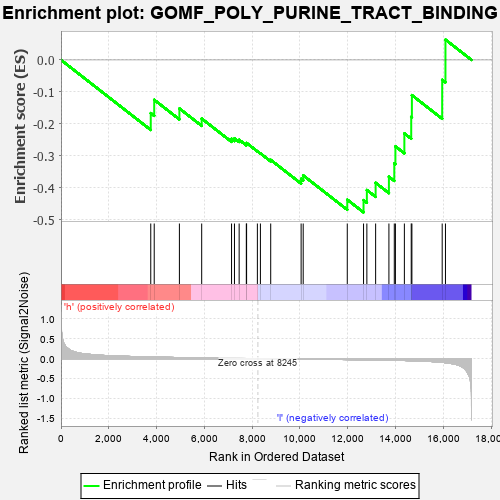

Supplement: Supplementary file 1 [file DataSheet_1.zip › enplot_GOMF_POLY_PURINE_TRACT_BINDING_489.png]

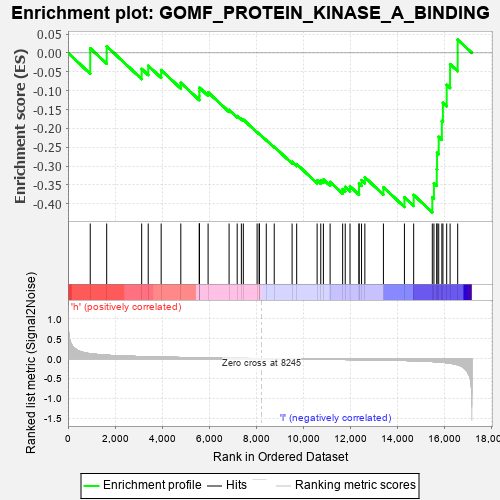

Supplement: Supplementary file 1 [file DataSheet_1.zip › enplot_GOMF_PROTEIN_KINASE_A_BINDING_528.png]

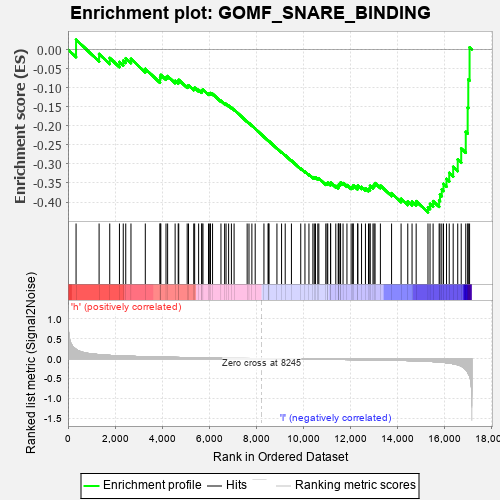

Supplement: Supplementary file 1 [file DataSheet_1.zip › enplot_GOMF_SNARE_BINDING_591.png]

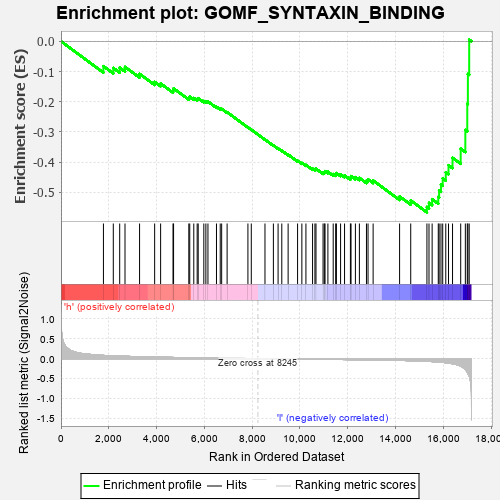

Supplement: Supplementary file 1 [file DataSheet_1.zip › enplot_GOMF_SYNTAXIN_BINDING_333.png]

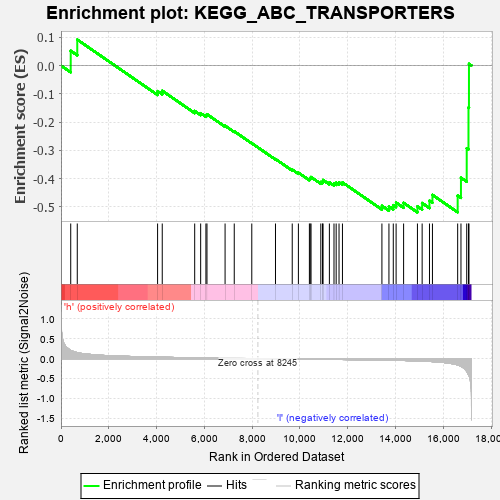

Supplement: Supplementary file 2 [file DataSheet_2.zip › enplot_KEGG_ABC_TRANSPORTERS_294.png]

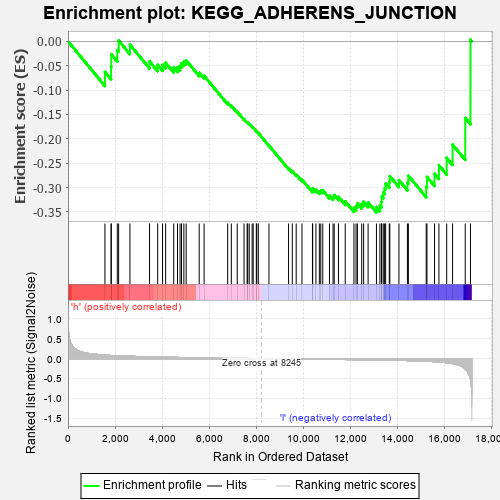

Supplement: Supplementary file 2 [file DataSheet_2.zip › enplot_KEGG_ADHERENS_JUNCTION_300.png]

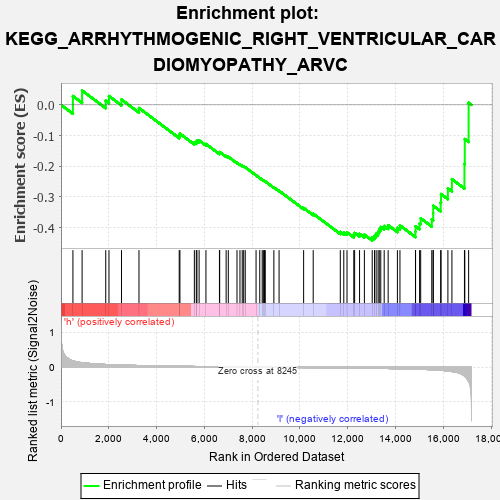

Supplement: Supplementary file 2 [file DataSheet_2.zip › enplot_KEGG_ARRHYTHMOGENIC_RIGHT_VENTRICULAR_CARDIOMYOPATHY_ARVC_312.png]

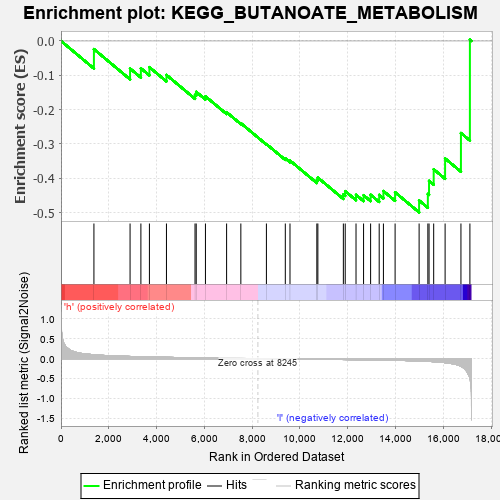

Supplement: Supplementary file 2 [file DataSheet_2.zip › enplot_KEGG_BUTANOATE_METABOLISM_288.png]

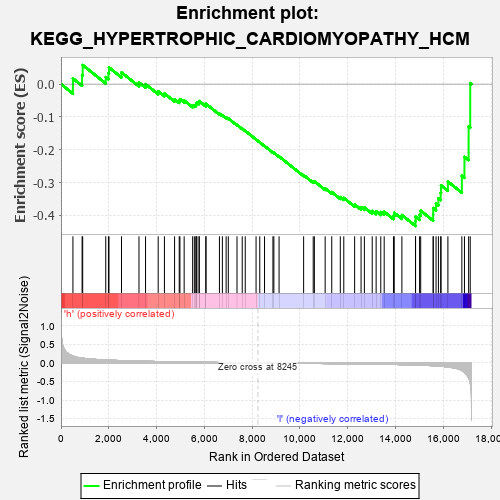

Supplement: Supplementary file 2 [file DataSheet_2.zip › enplot_KEGG_HYPERTROPHIC_CARDIOMYOPATHY_HCM_357.png]

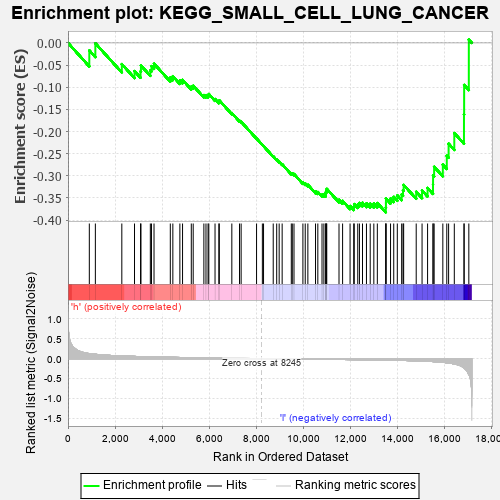

Supplement: Supplementary file 2 [file DataSheet_2.zip › enplot_KEGG_SMALL_CELL_LUNG_CANCER_291.png]

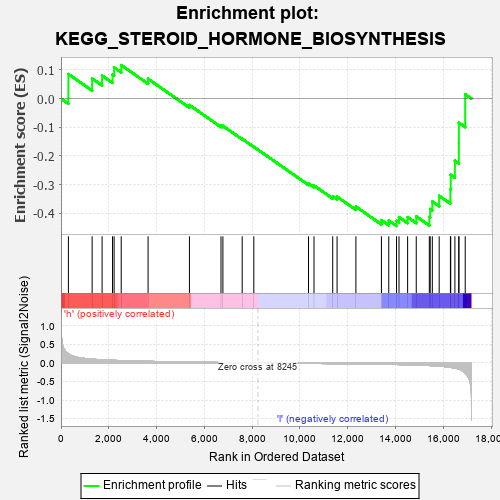

Supplement: Supplementary file 2 [file DataSheet_2.zip › enplot_KEGG_STEROID_HORMONE_BIOSYNTHESIS_363.png]
